# Development and validation of the Methotrexate Experience Questionnaire, a new methotrexate oral treatment adherence tool in rheumatoid arthritis

**DOI:** 10.1186/s41687-021-00339-5

**Published:** 2021-08-09

**Authors:** Jeffrey R. Curtis, Jan Michael Nebesky, Elodie de Bock, Christine de la Loge, Benoit Arnould, Robert Davey, Jenny Devenport, Attila Pethö-Schramm

**Affiliations:** 1 Division of Clinical Immunology and Rheumatology, University of Alabama at Birmingham, FOT 802, 510 20th Street South, Birmingham, AL 35294 USA; 2 Pharmaceuticals Division, F. Hoffmann-La Roche, Basel, Switzerland; 3 Patient-Centred Outcomes, ICON plc, Lyon, France; 4 eClinicalHealth Limited, Stirling, Scotland UK

**Keywords:** Rheumatoid arthritis, Patient-reported outcomes, Psychometrics, Adherence

## Abstract

**Objective:**

Despite the development of new biologic therapies, methotrexate (MTX) remains the preferred initial disease-modifying anti-rheumatic drug to treat rheumatoid arthritis (RA). Adherence to disease-modifying anti-rheumatic drugs is suspected to be highly variable potentially leading to reduced treatment effectiveness. This work aimed to develop and validate the Methotrexate Experience Questionnaire (MEQ), a tool to identify and characterize non-adherence to oral MTX.

**Methods:**

MEQ development included a literature review and qualitative interviews with RA patients and physicians in the United States. A retrospective, cross-sectional study using data from Optimum Patient Care Research Database, a large primary care database of electronic medical records in the United Kingdom, was conducted to finalize the MEQ and evaluate its psychometric properties.

**Results:**

Three hundred seven e-consented subjects (66% women, mean age of 65 years) completed the MEQ remotely, and were included in this analysis. Item-convergent and divergent validity were generally supportive of the construct validity of the MEQ and Cronbach’s alpha of 0.87 supported its reliability. The MEQ Total score presented statistically significant correlations of small to medium size with all selected concurrent scales, as expected; the highest correlation was obtained between the general acceptance score of ACCEPT and the MEQ Total score (0.55, *p* < 0.001). Known-groups validity was demonstrated as a logical pattern of higher MEQ scores was obtained for patients considered adherent with both the 6- and 12-month Proportion of Days Covered (mean MEQ total score 82.7 for 12-month PDC ≥ 80% against 76.3 for 12-month PDC < 80%, *p*< 0.0001). Additionally, a pattern of lower MEQ scores was obtained for patients with more severe disease assessed with Routine Assessment of Patient Index Data 3.

**Conclusion:**

The 24-item MEQ is a reliable and valid instrument to assess the adherence of RA patients taking MTX, potentially improving over historical refill rate metrics by providing insights into the individual reasons for lack of adherence. This information should facilitate clinician-patient discussions and help inform treatment decisions.

**Supplementary Information:**

The online version contains supplementary material available at 10.1186/s41687-021-00339-5.

## Background

Over the last decade, major progress has been made in the treatment of rheumatoid arthritis (RA) with the development of new biologic and targeted therapies recommended for use at later stages of the sequential RA treatment algorithm [1]. However, for the initial therapy of patients with RA conventional synthetic disease-modifying anti-rheumatic drugs (csDMARDs) are still the standard of care and methotrexate (MTX) remains the initial preferred csDMARD [2]. MTX can be administered orally, intravenously, intramuscularly, or self-administered subcutaneously. As with other csDMARDs, MTX may lead to undesired side effects contributing to suboptimal adherence with patients discontinuing therapy to avoid these side effects. At one year, up to half of RA patients discontinue MTX [3] and MTX adherence and persistence measured with a wide variety of different tools appears highly variable in patients with RA [4, 5]. Identifying the drivers of non-adherence to csDMARDs is essential to improve disease management. Indeed, inadequate or poor adherence reduces the effectiveness of treatment, which may lead to complications and deterioration in patients’ health and well-being.

A number of critical factors influence patient adherence to therapies, including patient-related factors (e.g. gender, age, affordability of care but also self-efficacy, beliefs, perceptions, motivations, and expectations), condition-related factors (e.g. severity of illness, duration), therapy-related factors (e.g. regiment complexity, adverse effects) and the quality of communication between patient and physician [6]. Therefore, physicians need to better understand patients’ needs and preferences and patients should be able to communicate as a partner with their physicians about treatment options and the evaluation of treatment effects [7].

Different tools designed to assess medication adherence are used in RA studies. There are generic tools such as the Morisky Medication Adherence Scale (MMAS), a self-reported adherence tool [8]; the Medication Adherence Report Scale (MARS), developed to assess medical compliance behavior among psychiatric patients [9]; the Beliefs About Medication Questionnaire (BMQ), asking patients about their beliefs about medications in general as well as questions about specific treatments that they have been prescribed [10]; and others, such as the Adherence Starts with Knowledge (ASK) questionnaire [11]. Additionally, there are RA-specific tools, such as the Compliance Questionnaire for Rheumatology (CQR) [12], the Medication Adherence Report Scale specific to RA (MARS-9RA) [13] and the Rheumatology Attitudes Index (RAI) [14]. However, none of these RA-specific tools is specific to adherence to any single drug, but rather, aggregates the behaviors and attitudes for all RA drugs together. Given the particular importance of MTX to the management of RA and other conditions (e.g. psoriatic arthritis), there is a need for a screening tool for use in standard care to assess adherence to MTX treatment and capture patients’ behaviors towards and experiences with MTX and thus improve patient management. This article describes the development and validation of the Methotrexate Experience Questionnaire (MEQ) for use in clinical practice to capture patient experience with oral MTX in RA and identify and characterize non-adherence to oral MTX.

## Patients and methods

The development and validation of the MEQ occurred in multiple stages: 1) Qualitative interviews to draft and pilot test the new measure, 2) MEQ administration and data collection to finalize and document its psychometric properties.

The qualitative research study and the psychometric validation study were independently submitted to and approved by the United States (US) Copernicus Group Independent Review Board and United Kingdom Health Research Authority, respectively. Written informed consent was given by all patients in the qualitative study to participate in the interviews. E-consent was given by all patients in the validation study to participate in the survey and have their prescription data used to derive a variable against which their survey data could be compared. The studies were conducted in compliance with the ethical principles derived from the Declaration of Helsinki.

### Development of the Methotrexate Experience Questionnaire

Questionnaire development followed the methodology recommended by the US Food and Drug Administration (FDA) for patient-reported outcome measures. First, a targeted literature search was performed to identify the questionnaires already existing that could be potential candidates for assessing adherence to MTX. The literature review also allowed the identification of the concepts of interest in relation with the purpose of the tool to be developed. Next, face-to-face concept elicitation interviews were conducted with adult patients with RA in the US. Patients were recruited through rheumatologists and were eligible if 18 years or older, diagnosed with RA according to the 1987 American College of Rheumatology-European League Against Rheumatism (ACR-EULAR) classification criteria, and currently or recently (within the past 12 months) treated with MTX. The objectives of these interviews were to understand patients’ experiences related to RA and its treatment (e.g., treatment constraints and difficulties, beliefs and perceptions, motivations or intentions to take or discontinue treatment). A study-specific interview guide was developed for the interviews, to provide the interviewer with questions to be asked to the patients during the interview and thus ensure coverage of the following themes during the interview: knowledge, beliefs and experience with RA, knowledge and experience about RA treatments, knowledge and experience with MTX (including experience with the mode of administration, impact of MTX on patients’ everyday lives, adherence), patients’ expectations and motivations towards MTX). Interviews were audio-recorded with patient’s permission. Recordings were transcribed verbatim and deidentified The analysis of the transcripts was performed using ATLAS.ti (Version 7.5.4; GmbH, Berlin, Germany [15]) according to grounded theory [16].

Based on an iterative and interpretative process, a model allowing visual representation of the factors influencing RA-treatment adherence and MTX adherence factors in RA patients was developed and informed the development of the draft MEQ. More specifically, the concepts selected to be measured in the questionnaire were discussed with an advisory board and comprised: physical barriers, financial barriers, practical barriers, emotional barriers, side-effects, perceptual barriers, and cognitive barriers. At least one item was generated for each concept above, using the wording collected during patient interviews.

Comprehension testing of the draft MEQ via face-to-face interviews was then performed with a new series of adult patients with RA in the US different than those who participated to the concept elicitation interviews, but meeting the same eligibility criteria. Patients completed the questionnaire and answered questions about its content, relevance and comprehension. In parallel, the draft MEQ was pilot tested by ten US rheumatologists with at least 5 years of practice as a rheumatologist regularly seeing patients with RA treated with MTX in consultation. Rheumatologists provided their feedback on the use of the draft questionnaire in clinical practice by completing the PRAgmatic Content and face validity-Test© (PRAC-Test©) [17], an evaluation questionnaire gathering physicians’ opinion and feedback on limitations and utility of a tool, as well as it ease of use and length and its use of the tool in clinical practice. Based on patients’ comments during the comprehension testing and clinicians’ feedback during pilot testing, the MEQ was revised.

### Finalization and psychometric validation of the methotrexate experience questionnaire

#### Study design and population

The psychometric validation study of the MEQ was conducted using a multi-center retrospective cross-sectional design. Data from Optimum Patient Care Research Database (OPCRD), a large United Kingdom primary care database of electronic medical records, were used to identify general practices that had high numbers of potentially eligible patients. Physicians from large general practices were invited to participate in the study to recruit patients meeting selection criteria. To be included, patients had to be aged 18 years or over at the date of the first MTX prescription in the database, be diagnosed with RA and have at least one recorded prescription for oral MTX to have occurred within 12 months and a second one within 6 months before screening the database. Patients were also to have at least 12-month history available in the OPCRD.

Eligible and interested patients were given access to an online platform to provide their e-consent and complete four questionnaires: (1) the MEQ, (2) the General Acceptance dimension of the Acceptance by the Patients of their Treatment (ACCEPT) questionnaire, (3) the Routine Assessment of Patient Index Data 3 (RAPID-3), and (4) a socio-demographic form. The electronic completion did not allow for missing data.

The ACCEPT General Acceptance dimension consists of three items (Advantages outweigh disadvantages, Medication acceptable solution and Medication worth taking in long-term) and its composite score ranges from 0 to 100, higher scores indicate greater medication acceptance [18]. RAPID-3 is a questionnaire allowing the derivation of a RA disease activity index score. The RAPID-3 includes three dimensions: patient functional status (FN), Pain (PN) and patient global assessment (PGA). Dimension scores range from 0 to 10, and the total score from 0 to 30, higher scores indicate higher RA activity [19]. For each patient, the physician extracted from their medical records additional information regarding their MTX treatment regimen, date of RA onset, concomitant medications and selected comorbidities. Physicians also answered the question “Does your patient take Methotrexate treatment for rheumatoid arthritis as recommended?” using the following response options: “Always or almost always (e.g. >80%)”, “Most of the time (e.g. 60-80%)”, “Sometimes (e.g. 20-60%)”, “Rarely (e.g. <20%)”, “I do not know”.

The Proportion of Days Covered (PDC) was used as the objective measure of adherence and anchor for the validation of the MEQ. PDC was calculated from the prescription data available in the OPCRD over a 6-month and a 12-month period preceding the completion of the patient forms. The PDC was calculated with the following equation: PDC = ((Number of days in period ‘covered’)/(Number of days in measurement period)) *100%. The average duration of the prescription in the database was 4.4 weeks. PDC ≥80%, the benchmark most commonly reported in the literature to define adherent patients [20] was used in this study.

#### Statistical analyses and psychometric properties

The initial structure of the questionnaire hypothesized following patient interviews was tested using Principal Component Analysis (PCA). The PCA aimed at evaluating whether empirical data confirms this hypothesized structure and defining adjustments that may be needed in the construction of the scoring algorithm. Several PCA were conducted on the MEQ items using VARIMAX rotation to refine the scale structure. The final structure was determined based on the hypothetical structure and using the Kaiser criterion. This analysis also allowed to identify items not fitting with the scale structure and possible candidates for exclusion. This data reduction analysis was run for the purpose of exploring the underlying factor structure of the latent variable “adherence”. A multitrait analysis was then used to confirm the final MEQ structure. In this analysis, Pearson correlation coefficients were calculated between each item and each dimension score after the considered item has been taken out. Two criteria were assessed: item convergent validity (correlation between each item and its own dimension ≥0.40) and item discriminant validity (each item should have a higher correlation with its own dimension than with other dimensions).

The internal consistency reliability of the MEQ (extent to which individual items are consistent with each other in the same dimension and reflect a single underlying concept) was assessed by the calculation of Cronbach’s alpha [21] for each dimension and overall. A minimum alpha value of 0.70 is generally considered acceptable [22]. Finally, construct validity was assessed by exploring the extent to which the MEQ scores relate to other variables based on the proximity in content between the two instruments (convergent validity) and the degree to which the MEQ can distinguish groups hypothesized to be different (known-groups validity). Convergent validity was assessed by calculating correlation coefficients between MEQ scores and the 6 and 12 months PDC, the General Acceptance score and RAPID-3 scores. For the known-groups validity, MEQ scores were described and compared between groups defined using 6 and 12 months PDC (PDC ≥ 80%; PDC < 80%; a priori hypothesis being that higher MEQ scores are expected for patients with PDC ≥ 80%), clinician rating of patient adherence (adherence > 80%; adherence ≤80%; a priori hypothesis being that higher MEQ scores are expected for patients with adherence≥80%), RA disease activity groups (High (RAPID-3 score > 12), Moderate (RAPID-3 score between 6.1 and 12), Low (RAPID-3 score between 3.1 and 6) and Near-remission (RAPID-3 score ≤ 3); a priori hypothesis being that higher MEQ scores are expected for patients with lower RAPID-3 scores meaning less severe disease) [23].

The discriminant ability of the MEQ Total score in classifying patients with low PDC was evaluated with the area under the Receiver Operating Characteristic (ROC) curve (AUC). The higher the AUC, the better the discrimination; with a value of 0.5 corresponding to discriminant ability no better than random guessing and 1 corresponding to perfect discriminant ability. The objective was then to select an optimal threshold on the MEQ Total score providing high sensitivity and sufficient specificity when confronted to the PDC binary criterion.

The threshold for statistical significance was fixed at 5%. Statistical analyses were performed using SAS software for Windows (Version SAS® Studio 3.7; SAS Institute, Inc., Cary, NC, USA).

## Results

### Development of the methotrexate experience questionnaire

**Fig. 1:**
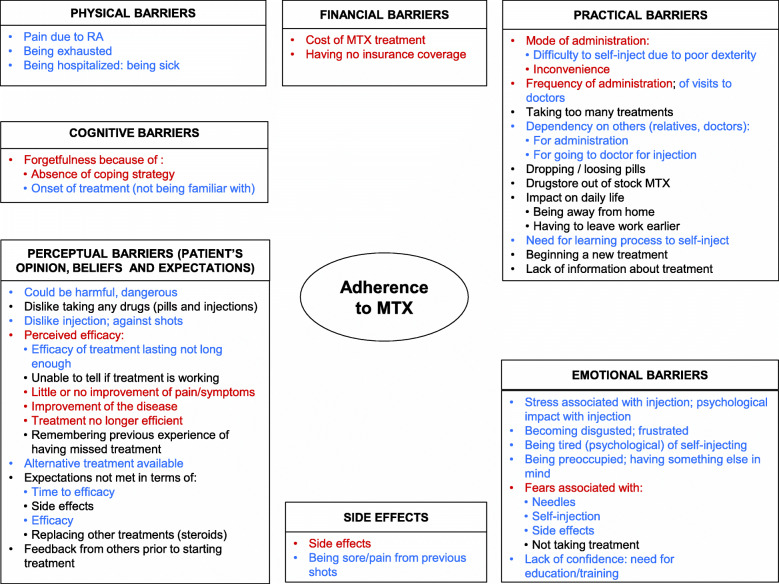
Conceptual model of the possible barriers specific to MTX adherence. In red, barriers to adherence reported by patients when talking about both injected and oral MTX, in black, barriers to adherence reported by patients when talking about oral MTX only, and in blue, barriers to adherence reported by patients when talking about injected MTX only

The qualitative research study was based on interviews conducted in 2014 with 32 patients (10 males/21 females/1 missing; median age 57 years) with RA (mean time since diagnosis 9.9 years). RA was mild for five patients, moderate for 21 and severe for eight. Five patients were treated with MTX alone, 21 were treated with MTX in combination with other RA medication(s), and six had stopped taking MTX. Amongst the factors and sub-factors identified during the interviews, some could either influence patients’ adherence in a negative way, and act as barriers to patients’ adherence, or in a positive way, and act as drivers to patients’ adherence, depending on the individual. As shown in Fig. 1, several factors associated with poor adherence to MTX were elicited from the analysis of the exploratory interviews: barriers (practical, physical, emotional, cognitive, financial), side effects, treatment perceived efficacy; doctor-related factors, patients’ beliefs, expectations and behaviors towards treatment, and external sources. It is to note that while patients reported some of these barriers when discussing about one mode of administration in particular, it is most likely that some of these barriers could also be attributed to a poor adherence in general, regardless of the mode of administration, Questions generic to both oral and injected MTX were generated using each patient’s own wording.

Eighteen patients (5 males/13 females; median age 61 years) with RA (mean time since diagnosis 8.7 years). participated to comprehension testing interviews. RA was mild for one patients, moderate for 11 and severe for six. Overall, patients understood and accepted the format and content of the questionnaire. Very little of the content was modified following this comprehension testing step. The following changes were made after agreement with clinical experts: i) reformulation of several items and of the section title from “Efficacy of MTX” to “Efficiency of MTX” to facilitate patient understanding; ii) Addition of two response choices, “Never” and “Always,” to the corresponding response scales, iii) Replacement of “Rheumatoid arthritis” by “arthritis” in the labels of items to make the questionnaire suitable for patients with other inflammatory arthritic diseases for which MTX is used, and iv) Addition of an area at the end of the questionnaire for open comments.

In parallel to the comprehension testing, ten rheumatologists recruited 30 patients treated with MTX for their RA and administered the MEQ during routine medical practice. Rheumatologists found the MEQ useful to monitor compliance and adherence, enhance communication with their patients; and help in therapeutic decision-making.

The resulting questionnaire included 29 items distributed across six hypothetical dimensions labelled according to their content coverage as: Convenience, Drivers of non-compliance, Benefits, Negative feelings, My opinion about my care, and General Experience with MTX. This version was included in the psychometric validation study.

### Finalization and psychometric validation of the methotrexate experience questionnaire

#### Population characteristics

From a total of 1387 potential subjects found in the OPCRD and loaded into the database, 1043 were deemed eligible and invited. A total of 317 patients were finally enrolled in the study, and 307 included in the analysis (4 patients for whom updated prescription history could not be obtained, and 6 patients with medical records quality issues preventing PDC calculation were excluded) As shown in Table 1, patients were mainly women (66%) and had a mean age of 65 years. The average time since diagnosis was 12 years and two thirds of the sample had been treated with MTX for more than 5 years. Using 6-month data, 89 (29%) participants were categorized as non-adherent (PDC < 80%), similarly using 12-month data, 85 (28%) participants were categorized as non-adherent.

**Table 1:** Characteristics of the patients included in the psychometric validation study

Characteristics	***N*** = 307
**Gender, n (%)**	
Female	203 (66.1%)
**Age (years), mean (SD)**	65.1 (12.5)
range	23.0–94.0
**Personal situation, n (%)**	
Living alone at home	58 (18.9%)
Living with other at home	248 (80.8%)
Living in assisted living environment	1 (0.3%)
Other	0 (0.0%)
**Education, n (%)**	
CGSE/O level	97 (31.6%)
A Level or equivalent	60 (19.5%)
Undergraduate degree	36 (11.7%)
Post-graduate	31 (10.1%)
Other	83 (27.0%)
**Professional status, n (%)**	
Full time paid employment	61 (19.9%)
Part time work	43 (14.0%)
Homemaker/housewife	17 (5.5%)
Student	1 (0.3%)
Unemployed	4 (1.3%)
Retired	171 (55.7%)
Other	10 (3.3%)
**Time from diagnosis to questionnaire completion (years), mean (SD)**	12.3 (9.8)
range	0.5–56.7
**Use of at least one RA treatment other than MTX, n (%)**	
Yes	220 (71.7%)
No	87 (28.3%)
**Presence of comorbidities, n (%)**	
Yes	133 (43.3%)
No	174 (56.7%)
**Time between first dose of MTX and questionnaire completion, n (%)**	
Less than a year	6 (2.0%)
From 1 to less than 5 years	94 (30.6%)
From 5 to less than 10 years	99 (32.2%)
10 years or more	108 (35.2%)
**Weekly MTX dosage (mg), mean (SD)**	15.7 (5.4)
range	5.0–30.0
**6-month PDC < 80%, n(%)**	89 (29.0%)
**12-month PDC < 80%, n(%)**	85 (27.7%)
**ACCEPT General Acceptance Score, mean (SD)**	74.2 (27.5)
range	0.0–100
**RAPID-3 Total Score, mean (SD)**	9.3 (5.9)
range	0.0–25.7

The mean ACCEPT general acceptance score was 74.2 (standard deviation, SD = 27.5), indicating that patients presented a good acceptance of their medication overall. The mean RAPID-3 total score was 9.3 (SD = 5.9), indicating moderate disease activity of RA on average, although values varied across patients, ranging from 0 (remission) to 25.7 (high disease activity).

#### Finalization of the MEQ

Iterative PCAs were performed to test the impact of variations in the number of factors on the scale structure (PCAs with number of factors free and then forced with defined number of factors (e.g., 4 and 5) were tested). Results from the PCA with five factors yielded the most readily interpretable dimensions for the MEQ structure and were used to define the final MEQ structure. The PCA results led to propose a final MEQ structure made of five multi-item dimensions (Convenience, Drivers of non-compliance, Benefits, Negative feelings, and My opinion about my care) close to the initial hypothesized structure which was based on the results of the qualitative interview analysis. The General Experience with MTX item was kept as a single item global assessment score. Given the drop in eigenvalues between the first (7.37) and the second factor (2.52), it was felt appropriate to calculate a total score in addition to the dimension scores. In the subsequent multitrait analysis, all multi-item dimensions, except for the Drivers of non-compliance dimension, presented good item-convergent validity as correlation between items and their own dimension (after exclusion of the considered item) were superior to 0.4. For all items, the item-divergent validity criterion was met. Five items presented significant issues, including ceiling effect, low item convergent validity and/or were redundant to other better fitting items. These items were removed from the final MEQ which includes 24 items rated on 4-point Likert scales. The content of the selected 24 items is presented in Table 2, and a review copy of the MEQ along with a scoring example is available in the supplementary files.

**Table 2:** Final structure of the MEQ and item-scale Pearson correlations from the multitrait analysis

Dimension		Item label	Pearson correlations^a^				
	Mean (SD) score		Benefits	Convenience	Drivers of non-compliance	Negative feelings	My opinion about my care
Benefits	71.6 (16.3)	MTX improves the symptoms of my arthritis	**0.79**	0.32	0.24	0.38	0.19
		I am sure that MTX is helping me	**0.76**	0.29	0.20	0.39	0.20
		The Benefits last until the next dose	**0.69**	0.25	0.30	0.36	0.15
		MTX meets my expectations because it works fast each time I take it	**0.71**	0.24	0.27	0.40	0.17
		I believe MTX is helping me control my arthritis in the long-term	**0.64**	0.28	0.19	0.41	0.17
		Each time I take MTX, it works quickly	**0.66**	0.27	0.28	0.33	0.13
		MTX meets my expectations because it helps relieve my arthritis symptom	**0.65**	0.23	0.29	0.45	0.17
Convenience	89.6 (13.8)	MTX is easy for me to take	0.31	**0.74**	0.28	0.29	0.01
		The number of times I have to take MTX is easy for me to remember	0.34	**0.71**	0.35	0.22	0.08
		MTX is part of my weekly routine	0.24	**0.65**	0.31	0.15	0.10
		MTX is affordable for me	0.22	**0.49**	0.26	0.17	−0.02
Drivers of non-compliance	93.4 (9.0)	When the symptoms of my arthritis improve, I skip taking MTX	0.09	0.24	***0.37***	0.12	0.02
		I postpone taking MTX, depending on the activities that I have planned	0.20	0.23	**0.43**	0.33	0.14
		When I am sick, I skip taking MTX	0.25	0.18	***0.35***	0.19	0.04
		When I am preoccupied or have many things on my mind, I forget to take MTX	0.19	0.31	**0.44**	0.26	0.15
		I skip taking MTX because of its side effects	0.18	0.11	***0.31***	0.22	0.15
		When I go on a trip, I forget to take MTX with me	0.15	0.17	***0.26***	0.05	0.04
		I need to be reminded to take MTX	0.20	0.24	***0.31***	0.18	0.13
Negative feelings	62.0 (21.7)	I am worried about the other problems (side effects) that MTX might cause me	0.38	0.18	0.23	**0.60**	0.27
		I am tired of taking MTX	0.41	0.22	0.31	**0.54**	0.26
		I have too many medications to take for my arthritis	0.39	0.22	0.22	**0.49**	0.24
My opinion about my care	82.2 (24.5)	My doctor involves me in decisions about my arthritis treatment	0.18	0.08	0.10	0.24	**0.45**
		I have enough information about MTX	0.18	−0.01	0.17	0.31	**0.45**
General Experience with MTX	77.7 (23.2)	Given my experience with MTX, I would prefer to continue taking it	–	–	–	–	–

Correlations indicating that items did not meet item convergent validity criterion are indicated in italics

Correlations indicating that items met item divergent validity criterion are indicated in bold

^a^Multitrait analysis performed only on multi-item dimensions

To score the MEQ, item responses were first coded so that a higher score corresponds to higher adherence, positive drivers of adherence or fewer barriers to adherence. Then a mean value of non-missing items within each scale for dimension score or of all non-missing items for the Total score was calculated, if at least 50% of the items were non-missing. The mean value was then linearly transformed so that it ranges from 0 to 100 with higher scores representing higher adherence, drivers of adherence or fewer barriers to adherence.

#### Psychometric validation of the final MEQ

Mean MEQ scores are shown in Table 2. The mean time to complete the MEQ was seven minutes.

Item-scale Pearson correlations from multitrait analyses performed on the final MEQ and presented in Table 2 support the instrument construct validity. Cronbach’s alpha for the MEQ Total score was 0.87, suggesting a good internal consistency reliability for the Total score. Benefits, Convenience and Negative feelings had Cronbach’s alpha of 0.89, 0.81 and 0.71, respectively, reflecting adequate internal consistency reliability. For the two other dimensions Drivers of non-compliance and My opinion about my care, the Cronbach’s alphas were 0.62, slightly below the 0.70 threshold.

As presented in Table 3, the MEQ Total score presented statistically significant correlations of small to medium size with all selected concurrent scales, as expected as these instruments measure related but distinct concepts. The highest correlation was obtained between the general acceptance score of ACCEPT and the MEQ Total score (0.55). At the dimension level, the highest correlations were obtained for the MEQ dimensions, Benefits and Negative feelings, and the general acceptance score of ACCEPT (0.49 and 0.46, respectively). Correlations between the MEQ subscale and Total scores and both the 6 and 12-month PDC were small (respectively 0.23 and 0.29 for the MEQ Total score), and systematically lower for the 6-month PDC.

**Table. 3:** Pearson correlation coefficients of MEQ scores with PDC and other concurrent scales scores

Score	6-month PDC	12-month PDC	ACCEPT Score	RAPID-3			
				Physical Function Score	Pain Score	Patient Global Assessment Score	Total Score
Benefits	0.18 **	0.21 ***	0.49 ***	− 0.21 ***	− 0.32 ***	− 0.27 ***	− 0.31 ***
Convenience	0.11	0.18 **	0.19 ***	0.02	−0.06	− 0.04	− 0.04
Drivers of non-compliance	0.25 ***	0.31 ***	0.20 ***	−0.13 *	−0.15 **	− 0.11	−0.15 *
Negative feelings	0.12 *	0.17 **	0.46 ***	−0.12 *	−0.17 **	− 0.17 **	−0.18 **
My opinion about my care	0.06	0.08	0.38 ***	−0.15 **	−0.17 **	− 0.19 ***	−0.19 ***
General Experience with MTX	0.15 **	0.22 ***	0.37 ***	−0.08	−0.11	− 0.07	−0.10
MEQ Total score	0.23 ***	0.29 ***	0.55 ***	−0.19 ***	−0.29 ***	− 0.25 ***	−0.28 ***

In bold, Correlations ≥0.4; in italics, 0.2 ≤ Correlations < 0.4

* *p* ≤ 0.05, ** *p* ≤ 0.01, *** *p* ≤ 0.001

**Fig. 2:**
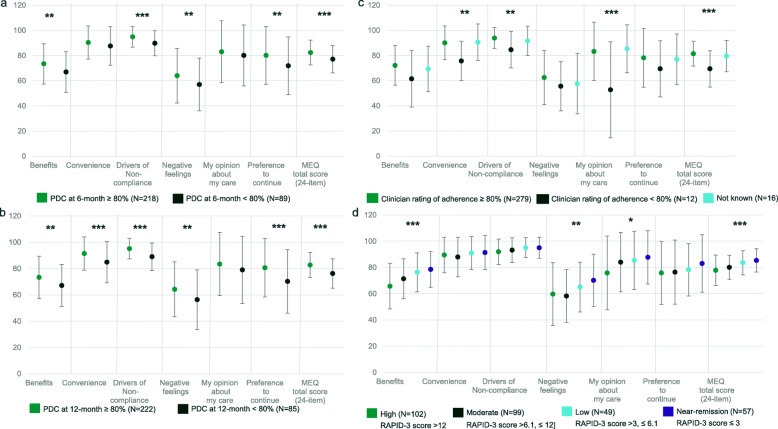
Known-groups validity - Mean (standard deviation) MEQ dimension and Total Scores by a) 6-month PDC, b) 12-month PDC, c) clinician rating of adherence and d) disease severity according to RAPID-3 scores. *p*-value from T-test for between-group comparisons when two groups, from ANOVA when more than two groups, * *p* ≤ 0.05, ** *p* ≤ 0.01, *** *p* ≤ 0.001

**Fig. 3:**
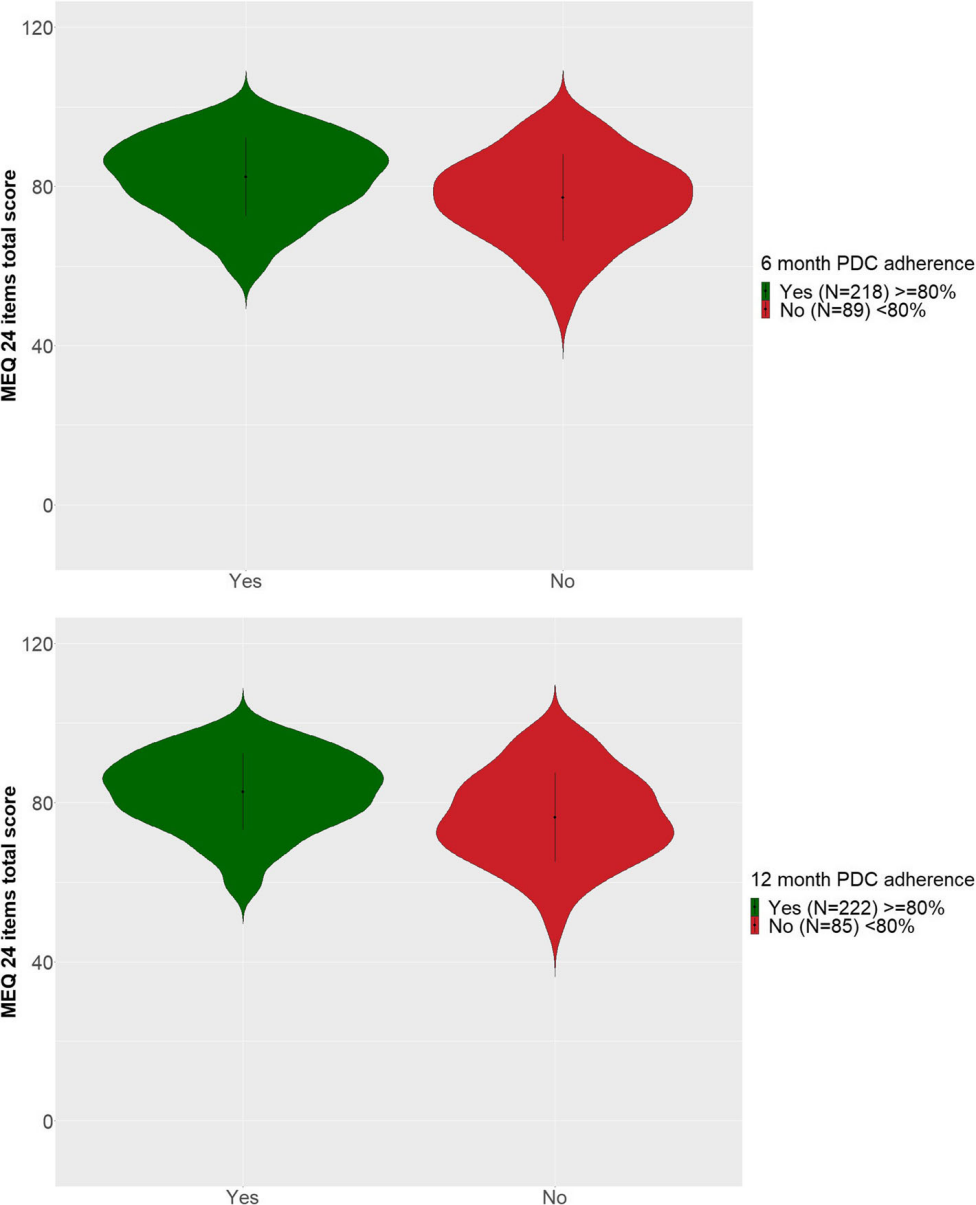
Known-groups validity – Violin plot showing the Mean, standard deviation and distribution of the MEQ Total Score by a) 6-month PDC and b) 12-month PDC

The MEQ demonstrated a good ability to discriminate between different groups expected to present different levels of adherence as shown in Figs. 2 and 3, and supplementary files. Patients with a high 6-month PDC (≥ 80%) presented a logical pattern of higher means in MEQ scores. The most significant between group differences (*p* < 0.001) were obtained for Drivers of non-compliance and for the MEQ Total scores. Results for the 12-month PDC were comparable to those obtained for the 6-month PDC, although between-group differences were generally more significant. Patients rated by their physicians as adherent (> 80%) also presented a logical pattern of higher means in MEQ scores, although these results were obtained from very few patients (12 out of 307, 3.9% were rated as non-adherent by their physician). Finally, patients with increasing disease severity as defined from RAPID-3 presented a trend of decreasing means in MEQ scores (i.e., decreasing adherence levels).

The AUC of the 6 and 12-month PDC (0.64 and 0.67 respectively) were modest revealing the limited ability of the MEQ to discriminate between the PDC adherence criteria used in this study. A threshold of 75 on the MEQ Total score to classify patients as adherent/non-adherent (adherent patients being those presenting an MEQ Total score ≥ 75) provided a sensitivity to detect non-adherence of 0.46 at 12 months (0.38 at 6 months) and a specificity of 0.81 at 12 months (0.78 at 6 months). With such threshold, 26.4% of patients were classified as non-adherent.

## Discussion

The MEQ was developed to assess adherence of patients taking MTX for RA and to obtain a patient adherence profile that could inform shared decision-making. The MEQ was finalized and further validated in patients taking oral MTX. The MEQ provides a comprehensive multi-dimensional assessment of patient adherence to MTX. It captures reasons for non-adherence (e.g., side effects, practical aspects related to MTX) allowing an adequate discussion between the physician and the patient around barriers encountered by the patient and what could be done to improve the patient adherence to the prescribed treatment regimen.

Results from the psychometric analyses conducted on the final MEQ were overall supportive of a valid and reliable instrument. The slightly poorer performance of the Drivers of non-compliance dimension is due to the composite nature of this dimension, which covers the different reasons why a patient may have missed or skipped a treatment intake, which are not expected to occur together, and the limited variability seen for many of these items.

As seen in this study, the correlations between PDC (6 and 12-month) and the MEQ dimensions were of limited amplitude. This may be explained by fundamental differences between the two measures. The PDC is a synthetic coarse measure built on a 6 or 12-month period and provides a retrospective quantification of patient adherence. It presents the appealing feature of being directly derivable from claim and prescription data, knowing that obtaining medication does not systematically mean taking medication. Also, the PDC covers only partial information about patient adherence to their treatment regimen and is sometimes referred as a measure of persistence [24]. The MEQ can be seen as a more thorough measure of adherence. It quantifies not only objective adherence behaviors (Drivers of non-compliance which presented the highest level of association with the PDC), but also other important components. Such components which were cited by patients in qualitative research, were to influence their adherence to their prescribed treatment regimen (e.g., perceived benefit they get from their treatment, convenience aspects, negative feelings experienced in the long-term and the opinion they have about their care).

The logical but limited convergence between the PDC and the MEQ has led to modest ability of the MEQ in discriminating between the PDC binary variables as could be seen with area under the ROC curves being lower than 0.7, as well as limited sensitivity and specificity for the proposed threshold of 75 on the MEQ Total score. Future work using a better anchor is needed to confirm the appropriateness of this preliminary cut-off of 75. PDC seemed a good anchor as it is one of the most used measures of adherence and it presented the appealing feature of being directly derivable from prescription data. Alternative study design using electronic pill dispensing boxes may provide a more sophisticated and reliable measure of adherence but would imply a higher burden and increased study costs and perhaps more importantly, create bias due to the Hawthorne effect [25] where behavior is altered due to observation and measurement.

While the 24-item MEQ provides a comprehensive profile of patients’ adherence behaviors and associated barriers and drivers of adherence, a shorter form is being explored to provide a brief synthetic summary measure of adherence with a limited burden on patients. Current efforts, which shall be published in a separate article, led to an 11-item short-form MEQ out of which a single total score can be derived. The analysis of the psychometric properties of this MEQ Short Form were overall supportive, and its discriminant ability was comparable to the original MEQ. Nonetheless, the original 24-item MEQ is key to inform in-depth discussion between physicians and patients. Shorter generic or RA-specific questionnaires exist and may be used to capture adherence, such as the MMAS [8], MARS [9], or the newly developed NIH Patient-Reported Outcomes Measurement Information System (PROMIS) Medication Adherence Scale (PMAS) [26], however the MEQ shall be used beyond the assessment of the adherence of patients taking MTX in RA and shall be used in daily medical practice to discuss specifics linked to MTX and allow for better-informed joint treatment decisions between physicians and their patients.

A limitation of this research relates to its retrospective study design. The absence of prospective data collection did not allow to test the ability of the MEQ in predicting future adherence. Similarly, the results obtained with the ROC curves allowed to provide a broad picture. However, these results need to be refined using retrospective and prospective data. Future prospective studies should thus be conducted to demonstrate the ability of the MEQ to predict adherence behaviors. We also recognize that some of the items (e.g. asking about side effects) might benefit from greater specificity, given that there are somewhat unique side effects associated with MTX that may be particularly bothersome to patients that are not observed with other RA therapies. While electronic implementations of the MEQ instrument likely would benefit from conditional branching if a patient selects an items where ‘side effects’ are mentioned to query about specifics (e.g. malaise, nausea, oral ulcers, alopecia), our intent was to minimize patient burden and minimize the instrument length. Another limitation is the response rate to the invitation to participate in the study of 30%, which might have led to enrolling fewer non-adherent patients. Indeed, most patients, approximately 70%, were highly adherent restraining the variability for the MEQ to discriminate between adherent and non-adherent patients. A last limitation is the fact that the questionnaire was validated in the United Kingdom. Local specificities in particular around insurance coverage, patient management may not allow for the generalisability of the findings to other countries. Future international research should be conducted to draw robust conclusion on the generalisability of the performance of the instrument.

## Conclusions

In conclusion, the MEQ appears to be a valid instrument to assess the adherence of patients taking MTX in RA and should help clinicians in daily medical practice to guide their discussions and make better-informed joint treatment decisions with their patients. Collecting patient experience with MTX in a systematic and standardized way using the MEQ will likely contribute to the recommended shared decision-making (SDM) process which is advocated in rheumatoid arthritis (RA), although not always observed in clinical practice [27]. Using both the PDC and the MEQ to assess patients’ adherence in research studies would provide complementary information and would be in line with a recent review concluding on the absence of a gold standard to assess adherence and recommending the use of at least two methods to capture adherence [28].

## Supplementary Information

**Additional file 1.**

**Additional file 2.**

## Data Availability

Data are available from the corresponding author upon reasonable request.

## Abbreviations

ACCEPT: Acceptance by the Patients of their Treatment questionnaire
MEQ: Methotrexate Experience Questionnaire
MTX: Methotrexate
PDC: Proportion of days covered
RA: Rheumatoid arthritis
RAPID-3: the Routine Assessment of Patient Index Data 3
