# Supplementary material for: Development and validation of the Methotrexate Experience Questionnaire, a new methotrexate oral treatment adherence tool in rheumatoid arthritis
Source: J Patient Rep Outcomes. 2021 Aug 9;5:69. doi: 10.1186/s41687-021-00339-5 (PMC8353039; doi:10.1186/s41687-021-00339-5)
Supplement: Supplementary file 1 — Additional file 1. [file 41687_2021_339_MOESM1_ESM.docx]

**Supplementary material**

***Instructions to clinicians and clinical staff***

The Methotrexate Experience Survey is intended for rheumatoid arthritis patients who are believed to be treated with methotrexate.

For example, this survey would be relevant for all patients who were on methotrexate at their last clinic visit. It is intended to be given to patients at each office visit, and administered before patients see their doctor. In most settings, this will occur in the waiting room or the examination room, prior to evaluation.

If you believe or know your patient is taking methotrexate, then please give them this survey to complete

### Methotrexate Experience Questionnaire

/__/__/ /__/__/ /__/__/__/__/

Day Month Year

This questionnaire is designed to help us know about your experience regarding your past or current use of methotrexate.

This information will help your rheumatologist make informed decisions about your current treatment for arthritis. This questionnaire is intended to be filled out at each office visit.

Please fill out this questionnaire in a quiet area and, if possible without assistance. If you do not know how to answer, please choose the response that best applies to you. There are no “right” or “wrong” answers. Please take all the time you need.

**Thank you**

**PRIOR TO COMPLETING THE QUESTIONNAIRE,**

**PLEASE ANSWER THE QUESTIONS BELOW**

- **Are you currently taking methotrexate?**

🞏 Yes 🞏 No

- **Are you taking methotrexate:**

🞏 By mouth, or

🞏 By injection?

- **In the last four weeks, how many doses of methotrexate have you taken? Consider all methotrexate taken within 24 hours as one dose (consider one dose as all methotrexate taken within 24 hours).**

🞏 None in the last four weeks

🞏 1 dose in the last four weeks

🞏 2 doses in the last four weeks

🞏 3 doses in the last four weeks

🞏 4 doses in the last four weeks

🞏 More than 4 doses in the last four weeks

***If you are no longer taking methotrexate, please remember back to when you were taking it when answering the questions below.***

**Methotrexate convenience aspect**

1. **Methotrexate is part of my weekly routine.**

| I strongly agree | I agree | I disagree | I strongly disagree |
| --- | --- | --- | --- |
| o | o | o | o |

1. **Methotrexate is easy for me to take.**

| I strongly agree | I agree | I disagree | I strongly disagree |
| --- | --- | --- | --- |
| o | o | o | o |

1. **The number of times I have to take methotrexate is easy for me to remember.**

| I strongly agree | I agree | I disagree | I strongly disagree |
| --- | --- | --- | --- |
| o | o | o | o |

1. **Methotrexate is affordable for me.**

| I strongly agree | I agree | I disagree | I strongly disagree |
| --- | --- | --- | --- |
| o | o | o | o |

***If you are no longer taking methotrexate, please remember back to when you were taking it when answering the questions below.***

**Non-compliance drivers**

1. **When I go on a trip, I forget to take methotrexate with me.**

| Always | Most of the time | Sometimes | Never |
| --- | --- | --- | --- |
| o | o | o | o |

1. **When I am sick, I skip taking methotrexate.**

| Always | Most of the time | Sometimes | Never |
| --- | --- | --- | --- |
| o | o | o | o |

1. **When the symptoms of my arthritis improve, I skip taking methotrexate.**

| Always | Most of the time | Sometimes | Never |
| --- | --- | --- | --- |
| o | o | o | o |

1. **When I am preoccupied or have many things on my mind, I forget to take methotrexate.**

| Always | Most of the time | Sometimes | Never |
| --- | --- | --- | --- |
| o | o | o | o |

1. **I postpone taking methotrexate, depending on the activities that I have planned.**

| Always | Most of the time | Sometimes | Never |
| --- | --- | --- | --- |
| o | o | o | o |

1. **I need to be reminded to take methotrexate.**

| Always | Most of the time | Sometimes | Never |
| --- | --- | --- | --- |
| o | o | o | o |

1. **I skip taking methotrexate because of its side effects.**

| I am currently not taking methotrexate because of its side effects | I am currently not taking methotrexate because of other reasons | Always | | Most of the time | | Sometimes | | Never | | |
| --- | --- | --- | --- | --- | --- | --- | --- | --- | --- | --- |
| o | o | | o | | o | | o | | o |  |

***If you are no longer taking methotrexate, please remember back to when you were taking it when answering the questions below.***

**BENEFITS and expectations**

1. **I am sure that methotrexate is helping me.**

| I strongly agree | I agree | I disagree | I strongly disagree |
| --- | --- | --- | --- |
| o | o | o | o |

1. **Methotrexate improves the symptoms of my arthritis.**

| I strongly agree | I agree | I disagree | I strongly disagree |
| --- | --- | --- | --- |
| o | o | o | o |

1. **The benefits of methotrexate last until the next dose.**

| I strongly agree | I agree | I disagree | I strongly disagree |
| --- | --- | --- | --- |
| o | o | o | o |

1. **Each time I take methotrexate, it works quickly.**

| I strongly agree | I agree | I disagree | I strongly disagree |
| --- | --- | --- | --- |
| o | o | o | o |

1. **I believe methotrexate is helping me control my arthritis in the long term.**

| I strongly agree | I agree | I disagree | I strongly disagree |
| --- | --- | --- | --- |
| o | o | o | o |

1. **Methotrexate meets my expectations because it helps relieve my arthritis symptom.**

| Exceeds my expectations | Completely meets my expectations | Partially meets my expectations | Does not meet my expectations |
| --- | --- | --- | --- |
| o | o | o | o |

1. **Methotrexate meets my expectations because it works fast each time I take it.**

| Exceeds my expectations | Completely meets my expectations | Partially meets my expectations | Does not meet my expectations |
| --- | --- | --- | --- |
| o | o | o | o |

***If you are no longer taking methotrexate, please remember back to when you were taking it when answering the questions below.***

**My feelings about methotrexate**

1. **I am tired of taking methotrexate.**

| I strongly agree | I agree | I disagree | I strongly disagree |
| --- | --- | --- | --- |
| o | o | o | o |

1. **I am worried about the other problems (side effects) that methotrexate might cause me.**

| I strongly agree | I agree | I disagree | I strongly disagree |
| --- | --- | --- | --- |
| o | o | o | o |

1. **I have too many medications to take for my arthritis.**

| I strongly agree | I agree | I disagree | I strongly disagree |
| --- | --- | --- | --- |
| o | o | o | o |

***If you are no longer taking methotrexate, please remember back to when you were taking it when answering the questions below.***

**My opinion about my care**

1. **My doctor involves me in decisions about my arthritis treatment.**

| No, but I would like to be more involved in decisions | No, and I don’t want to be more involved in decisions | Yes, and I would like to be more involved in decisions | Yes, completely |
| --- | --- | --- | --- |
| o | o | o | o |

1. **I have enough information about methotrexate.**

| No,  but I would like to have more information | | No,  and I don’t want to have more information | | Yes,  but I would like to have even more information | | Yes, completely | |
| --- | --- | --- | --- | --- | --- | --- | --- |
| o | o | | o | | o | |  |

***If you are no longer taking methotrexate, please remember back to when you were taking it when answering the questions below.***

**MY METHOTREXATE TREATMENT IN GENERAL**

1. **Given my experience with methotrexate, I would prefer to continue taking it.**

| \| I strongly agree \| I agree \| I disagree \| I strongly disagree \| \| --- \| --- \| --- \| --- \| \| o \| o \| o \| o \| |
| --- | --- | --- | --- | --- | --- | --- | --- | --- |

| **Comments:** |
| --- |

Please check that you have answered all the questions.

Thank you for taking the time to answer these questions.

**RECODE OF ITEMS FOR SCORING ALGORITHM**

| Dimension | Item | Original values | Item Generated Score |
| --- | --- | --- | --- |
| MTX Convenience aspect | 1. Methotrexate is part of my weekly routine | 1=I Strongly Agree  2=I agree  3=I disagree  4=I Strongly Disagree | Item score = 5 – original value |
|  | 2. Methotrexate is easy for me to take |  |  |
|  | 3. The number of times I have to take Methotrexate is easy for me to remember |  |  |
|  | 4. Methotrexate is affordable for me |  |  |
| *Non-Compliance drivers* | 5. When I go on a trip, I forget to take MTX | 1=Always  2=Most of the time  3=Sometimes  4=Never | Item score = original value |
|  | 6. When I am sick, I skip taking MTX |  |  |
|  | 7. When the symptoms of my arthritis improve, I skip taking MTX |  |  |
|  | 8. When I am preoccupied or have many things on my mind, I forget to take MTX |  |  |
|  | 9. I postpone taking MTX, depending on the activities that I have planned |  |  |
|  | 10. I need to be reminded to take MTX |  |  |
|  | 11. I skip taking MTX because of its side effects | 5=I am currently not taking MTX because of its side effects | Item score=1 |
|  |  | 6=I am currently not taking MTX because of other reasons | Item score is missing |
|  |  | 1=Always | Item score = original value |
|  |  | 2=Most of the time |  |
|  |  | 3=Sometimes |  |
|  |  | 4=Never |  |
| Benefits and expectations | 12. I am sure that MTX is helping me | 1=I Strongly Agree  2=I agree  3=I disagree  4=I Strongly Disagree | Item score = 5 – original value |
|  | 13. MTX improves the symptoms of my arthritis |  |  |
|  | 14. The benefits of MTX last until the next dose |  |  |
|  | 15. Each time I take MTX, it works quickly |  |  |
|  | 16. I believe MTX is helping me control my arthritis in the long term |  |  |
|  | 17. MTX meets my expectations because it helps relieve my arthritis symptoms | 1=Exceeds my expectations  2=Completely meets my exp.  3=Partially Meets my exp.  4=Does not meet my exp. | Item score = 5 – original value |
|  | 18. MTX meets my expectations because it works fast each time I take it |  |  |
| My feelings about MTX | 19. I am tired of taking MTX | 1=I Strongly Agree  2=I agree  3=I disagree  4=I Strongly Disagree | Item score = original value |
|  | 20. I am worried about the other problems (side effects) that MTX might cause me |  |  |
|  | 21. I have too many medications to take for my arthritis |  |  |
| My opinion about my care | 22. My doctor involves me in decisions about my arthritis treatment | 1=No, but I would like to be more involved in decisions  2=No, and I don’t want to be more involved into decisions  3=Yes, and I would like to be more involved in decisions  4=Yes, completely | Item score = original value |
|  | 23. I have enough information about MTX | 1=No, but I would like to have more information  2=No, and I don’t want to have more information  3=Yes, but I would like to have more information  4=Yes, completely | Item score = original value |
| General Experience with MTX | 24. Given my experience with MTX, I would prefer to continue taking it | 1=I Strongly Agree  2=I agree  3=I disagree  4=I Strongly Disagree | Item score = 5 – original value |

**SCORING ALGORITHM**

The MEQ Total score ranges from 0 to 100 with higher scores indicating better adherence. This score can be calculated if at least 50% of the items of the MEQ are completed and is computed as follows:

$$MEQ total score= \frac{1}{3} \left( mean of all completed MEQ items-1 \right)\times100$$

The score of each dimension is computed using the same equation with “mean of all completed MEQ items” replaced by “mean of all completed MEQ items of the dimension”.
