# Supplementary material for: Development and validation of the Methotrexate Experience Questionnaire, a new methotrexate oral treatment adherence tool in rheumatoid arthritis
Source: J Patient Rep Outcomes. 2021 Aug 9;5:69. doi: 10.1186/s41687-021-00339-5 (PMC8353039; doi:10.1186/s41687-021-00339-5)

**Supplementary material**

**Supplementary Figure 1. Known-groups validity** – Radar plots showing mean MEQ dimension scores by a) 6-month PDC, b) 12-month PDC, c) clinician rating of adherence and d) disease severity according to RAPID-3 scores.


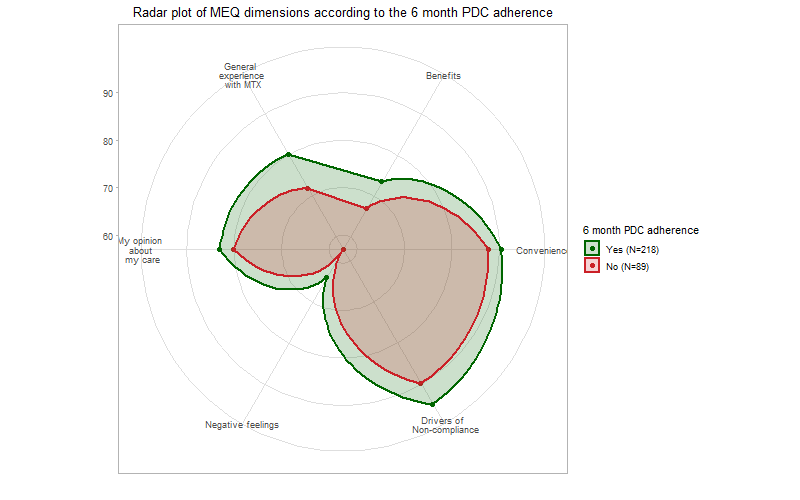
a)

b)


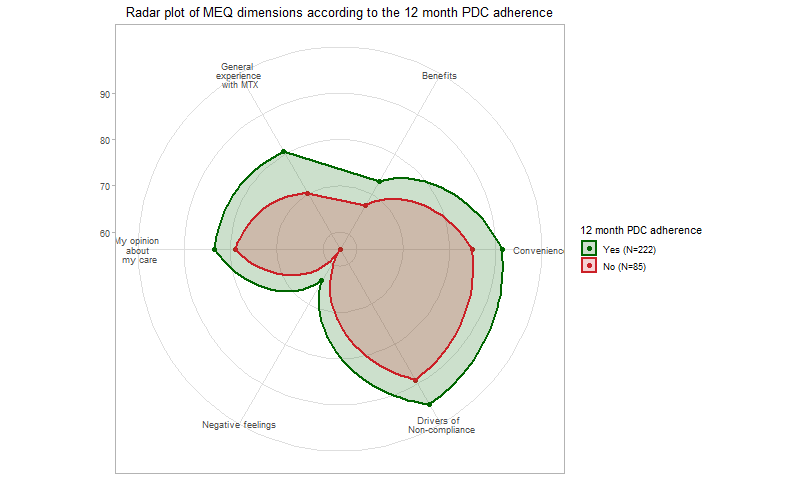


c)


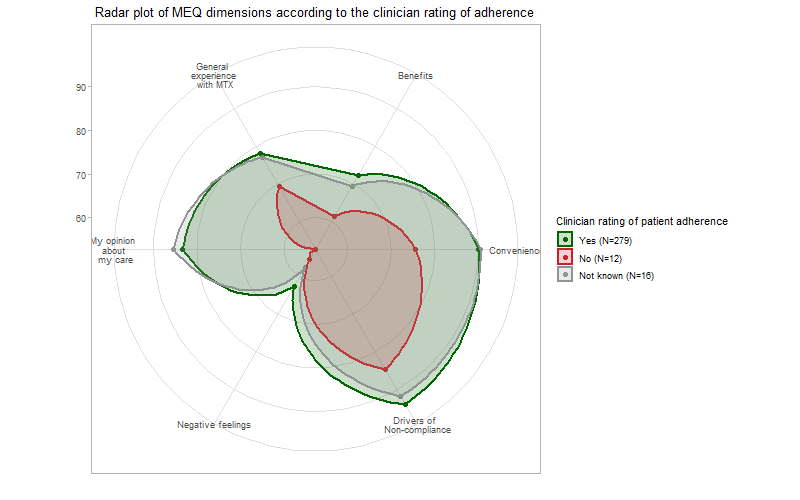


d)


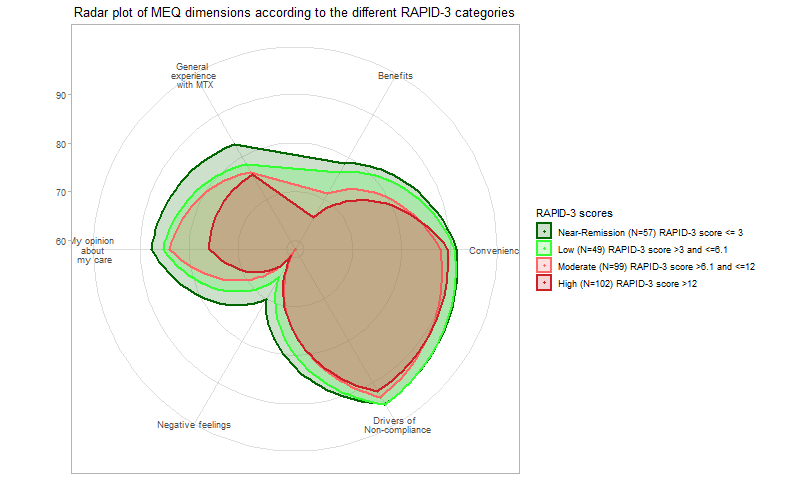

Supplement: Supplementary file 2 — Additional file 2. [file 41687_2021_339_MOESM2_ESM.docx]
